# Pathomechanisms of Paraneoplastic Myasthenia Gravis

**DOI:** 10.1080/10446670310001598528

**Published:** 2003-03

**Authors:** Philipp Ströbel, Tobias Preisshofen, Markus Helmreich, Hans Konrad Müller-Hermelink, Alexander Marx

**Affiliations:** Institute of Pathology University of Wuerzburg Josef-Schneider-Strasse 2 Wuerzburg 97080 Germany

## Abstract

Thymic T cell development is characterized by sequential selection processes to ensure generation of a self-tolerant, immuncompetent mature T cell repertoire. Malfunction of any of these selection processes may potentially result in either immunodeficiency or autoimmunity. Myasthenia gravis (MG) is a typical autoimmune manifestation of thymic epithelial tumors (thymomas) and is related to the capacity of these tumors to generate and export mature T cells. Analysis of the factors that lead to autoimmunization in thymomas will help to understand the mechanisms that prevent MG under physiological conditions in humans. In a comparison of MG(+) and MG(-) thymomas, we could show that only thymomas capable of generating mature CD45RA+CD4+ T cells are associated with MG (*p*< 0.0001), while terminal thymopoiesis was abrogated in MG(-) thymomas. In particular, acquisition of the CD27+CD45RA+ phenotype appears to be a critical checkpoint of late T cell development in the human thymus and may play an important role in the prevention of autoimmunity. Moreover, MG(-) thymomas were virtually depleted of regulatory (CD4+CD25+) T cells (regT), while regT were readily detectable in MG(+) thymomas, albeit at significantly reduced numbers compared to control thymuses. Thus, in MG(+) thymoma patients, thymectomy apparently also results in removal of a regulatory T cell pool and may explain the frequent temporary postoperative deterioration of MG in these patients.


*Clinical & Developmental Immunology*, March 2003, Vol. 10 (1), pp. 7–12

Taylor & Francis healthsciences

### Pathomechanisms of Paraneoplastic Myasthenia Gravis

PHILIPP STRÖBEL*, TOBIAS PREISSHOFEN, MARKUS HELMREICH, HANS KONRAD MÜLLER-HERMELINK and ALEXANDER MARX

*Institute of Pathology, University of Wuerzburg, Josef-Schneider-Strasse 2, 97080, Wuerzburg, Germany*

Thymic T cell development is characterized by sequential selection processes to ensure generation of a self-tolerant, immuncompetent mature T cell repertoire. Malfunction of any of these selection processes may potentially result in either immunodeficiency or autoimmunity. Myasthenia gravis (MG) is a typical autoimmune manifestation of thymic epithelial tumors (thymomas) and is related to the capacity of these tumors to generate and export mature T cells. Analysis of the factors that lead to autoimmunization in thymomas will help to understand the mechanisms that prevent MG under physiological conditions in humans. In a comparison of MG(+) and MG(−) thymomas, we could show that only thymomas capable of generating mature CD45RA+CD4+ T cells are associated with MG ($p < 0.0001$), while terminal thymopoiesis was abrogated in MG(−) thymomas. In particular, acquisition of the CD27+CD45RA+ phenotype appears to be a critical checkpoint of late T cell development in the human thymus and may play an important role in the prevention of autoimmunity. Moreover, MG(−) thymomas were virtually depleted of regulatory (CD4+CD25+) T cells (regT), while regT were readily detectable in MG(+) thymomas, albeit at significantly reduced numbers compared to control thymuses. Thus, in MG(+) thymoma patients, thymectomy apparently also results in removal of a regulatory T cell pool and may explain the frequent temporary postoperative deterioration of MG in these patients.

*Keywords*: Myasthenia gravis; Thymus; Thymoma; Human; T cell; Naive; CD4; Regulatory

### INTRODUCTION

Myasthenia gravis (MG) is an autoimmune disease mediated by anti-acetylcholine receptor autoantibodies that cause muscle weakness by impairing neuromuscular transmission (Lindstrom *et al.*, 1998). In a vast majority of MG patients, the thymus shows pathologic alterations that are believed to be aetiologically relevant for the disease. In a subgroup of patients, MG is a paraneoplastic manifestation of a thymic epithelial neoplasm (thymoma). The current WHO classification recognizes five MG-associated histological thymoma subtypes, that reflect not only morphological, but also functional and clinical differences among the different types. Among these, type AB, B2 and B3 thymomas are by far the most frequent subtypes and at the same time show the highest association with MG (Rosai, 1999; Muller-Hermelink and Marx, 2000). Importantly, MG-associated thymomas retain many functional features of the normal thymus, namely the capacity to generate and export mature T cells from immature precursors. However, no systematic differences that allow to distinguish between MG(+) and MG(−) thymomas have been identified so far with respect to T cell maturation. We and others have reported on impaired maturation almost exclusively of the CD4 lineage, while development of the CD8 lineage is better preserved (Nenninger *et al.*, 1998; Strobel *et al.*, 2001). Furthermore, all known thymocyte maturation stages in terms of CD3, CD69, CD4 and CD8 expression can be detected in virtually all MG(+) and MG(−) thymomas (Takeuchi *et al.*, 1995; Nenninger *et al.*, 1998). Recently, the introduction of measurement of so-called T cell receptor (TCR) rearrangement excision circles (TRECS) has allowed for the quantification of naive T cells and thus to directly assess the contribution of the thymus and supposedly the thymoma to the peripheral T cell pool (Douek *et al.*, 1998). Buckley *et al.* reported on abnormally increased numbers of naïve CD4+ and CD8+ T cells in the blood of MG(+) thymoma patients, while an increase only of naïve CD8+ T cells was detected in the blood of three MG(−) thymoma patients (Buckley *et al.*, 2001). This study suggested a role of thymoma-derived CD4+ T cells in the pathogenesis of paraneoplastic MG. However, it did not resolve the underlying pathophysiology at the level of intratumorous thymopoiesis. To address these questions, we analyzed the intratumorous generation of mature naive CD45RA+ thymocyte subsets (Vanhecke *et al.*, 1995) in thymomas

*Corresponding author. Tel.: +49-931-201-47878. Fax: +49-931-201-47440. E-mail: path036@mail.uni-wuerzburg.de

ISSN 1740-2522 print/ISSN 1740-2530 online © 2003 Taylor & Francis Ltd
DOI: 10.1080/10446670310001598528

TABLE I Clinical data of thymoma patients and control persons

| Patient Number | WHO thymoma subtype | Sex | Age (years) | MG |
|---|---|---|---|---|
| 1–9 | 2 AB; 7 B2/B3 | 6 F, 3 M | 39–75 | yes |
| 10–22 | 6 AB; 7 B2/B3 | 7 F, 6 M | 39–70 | no |
| Controls | | | | |
| Thymus; $n = 13$ | TFH | 10 F, 3 M | 8–37 | yes |

TFH, thymic follicular hyperplasia.

with and without associated MG. Intratumorous apoptosis was studied using TUNEL assays.

Our results show that presence of paraneoplastic MG is highly correlated with the efficiency of thymomas to produce and export mature naïve CD4 T cells. The transition from the CD27+CD45RA− to the CD27+CD45RA+ stage appears to be a critical checkpoint in terminal thymocyte maturation not only in thymomas, but also in the normal thymus. Efficient deletion at this stage may be an important mechanism of central tolerance and in the prevention of autoimmunity.

### MATERIALS AND METHODS

### Patients

The clinical data of the patients and control persons included in this study are summarized in Table I. Blood samples were taken at time of thymectomy. Only patients without steroid or immunsuppressive treatment prior to surgery were included in this study. The diagnosis of MG was based on clinical features, decrement testing on 3 Hz serial stimulation, and the detection of anti-AChR antibodies as described previously (Nenninger *et al.*, 1998). All MG(+) patients were autoantibody positive.

### Tumors and Cell Preparation

Thymomas were classified according to the WHO classification (Rosai, 1999).

Thirteen non-neoplastic thymuses with inflammatory changes (thymic lymphofollicular hyperplasia, TFH) were used as controls for the thymic compartment. Prior to staining for FACS analysis (see below), thymocytes and PBMNC from all samples were isolated by Ficoll-Hypaque density gradient centrifugation as described in detail previously (Nenninger *et al.*, 1998).

### Nomenclature of Naïve Thymocyte- and T Cell Subsets

Thymocytes expressing either CD4 or CD8 together with CD3, CD69, CD27 and CD45 RA were designated as “mature naïve CD4 or CD8 T cells”, respectively.

### Flow Cytometry

Sampling and data analyses of thymocytes and PBMNC were performed on a FACScan flow cytometer with Lysis II software (Becton Dickinson, Heidelberg, Germany) as described (Nenninger *et al.*, 1998). The CD3+ T-cell subset was gated for all analyses. $2 \times 10^5$ cells were stained for 3-colour FACS analysis with a panel of surface antigen-directed monoclonal antibodies (mAbs) as described previously (Nenninger *et al.*, 1998). The panel of antibodies included anti-CD3 labeled either with FITC (Sigma, Deisenhofen, Germany), PE (Dako, Hamburg, Germany) or TRI (Medac, Hamburg, Germany), anti-CD4 labeled with either PE or TRI, anti-CD8-PE (Dako); anti-CD69 (either FITC-or PE-labeled) (Becton-Dickinson); anti-CD27-PE (Pelicluster, Netherlands), CD45RO and anti-CD45RA (either FITC- or PE-labeled) (Dianova, Hamburg, Germany). Isotype controls were purchased from Sigma and Medac.

### TUNEL Assay

Thymocytes isolated from thymomas and from non-neoplastic control thymuses were analyzed using a commercially available TUNEL-kit (Boehringer, Germany) according to the manufacturer’s instructions. Prior to TUNEL labeling, cells were fixed and permeabilized using Cytofix/Cytoperm solution and Perm/Wash buffer (Pharmingen, Germany).

### Statistical Analyses

All statistical analyses were performed using Chi-Square and Man-Whitney-U test ($p$-value $< 0.05$, unless otherwise indicated) provided by the SPSS software (Version 2000, SPSS Inc., Chicago, U.S.). In all column graphs shown, the statistical mean ± standard error of the mean are depicted.

### RESULTS

### Acquisition of the Mature Naive CD4+ CD27+ CD45RA+ Phenotype is a Critical Checkpoint in Terminal Thymocyte Maturation

We and others have previously reported on impaired early T cell development in different thymoma subtypes (Takeuchi *et al.*, 1995; Nenninger *et al.*, 1998; Strobel *et al.*, 2001). Analyzing terminal T cell maturation in 13 non-neoplastic control thymuses, the population size of CD4+ T cells was cut down by 50–60% at the transition from the CD27+CD45RA− to the CD27+CD45RA+

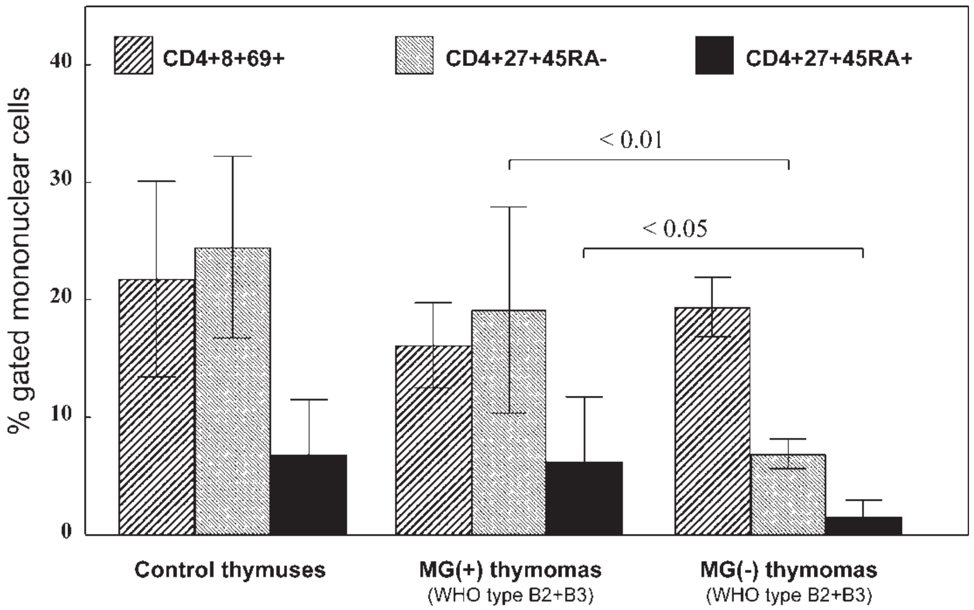

FIGURE 1 The percentage of mature naïve CD4 T cells is highly reduced in MG(−) thymomas. FACS analysis of naïve thymocyte subsets shows a marked, statistically significant reduction of CD4+CD27+ and of mature naive CD4+CD45RA+ T cells in MG(−) thymomas compared both to MG(+) thymomas and non-neoplastic control thymuses.

stage. The same was true for MG(+) thymomas, particularly of the histological subtypes B2 and B3, in which the earlier steps of T cell maturation appear to be largely undisturbed (Strobel *et al.*, 2001). By contrast, the proportion of CD4+CD27+CD45RA+ T cells was highly significantly reduced in all MG(−) thymomas analyzed compared to both MG(+) thymomas and control thymuses (4.3 ± 2.0 in MG(−) thymomas vs. 24.4 ± 18.9 in MG(+) thymomas, $p < 0.0001$; Fig. 1). Thus, on Chi-Square-test, presence of MG was significantly linked to high levels of mature naïve CD4+ T cells ($p < 0.01$). Preliminary results of a TUNEL analysis showed that the subtotal loss of the mature naive CD4 T cell subset is due to deletion/apoptosis (Fig. 2). A subset of both MG(+) and MG(−) thymomas also showed a striking reduction of mature naive CD8+ cells.

Together, these data point to the action of negatively selecting mechanisms at the transition from the CD27+CD45RA− to the CD27+CD45RA+ mature naive phenotype. Inefficiency of these negatively selecting mechanisms (as indicated by obviously permissive negative selection of CD4 cells in MG(+) thymomas) may cause break of central tolerance.

### Regulatory (CD4+ CD25+) T Cells are Highly Reduced in Thymomas

In recent years, a specific CD25+CD4+ T cell subpopulation with regulatory functions (regulatory T cells, regT) has been identified in the thymus of both mice and humans (Itoh *et al.*, 1999; Stephens *et al.*, 2001). Thymus-derived regT are believed to be generated by high-affinity interactions with self-peptides present in the thymus (Jordan *et al.*, 2001). Several studies showed that regT cells play an important role in controlling organ-specific autoimmunity (Roncarolo and Levings, 2000; Sakaguchi *et al.*, 2001; Shevach *et al.*, 2001). In a comparison of MG(+) and MG(−) thymomas with control thymuses, we found a statistically significant 5-fold reduction of regT cells in all thymomas irrespective of presence or absence of MG ($p < 0.05$; Fig. 3), although reduction of regT cells was more pronounced in MG(−) thymomas.

### DISCUSSION

In contrast to the well characterized early steps in thymocyte maturation (Berg and Kang, 2001; Hogquist, 2001; MacDonald *et al.*, 2001), the mechanisms controlling the late phase of thymopoiesis are poorly characterized. The crucial role of negative selection (i.e. elimination of the majority of autoreactive thymocytes) in the prevention of autoimmunity is a scientific paradigm established by many experimental models (Laufer *et al.*, 1996; 1999; Naquet *et al.*, 1999; Rubin and Kretz-Rommel, 2001). Earlier studies on terminal thymopoiesis in humans suggest (a) that late T cell development is an MHC-independent process (Vanhecke *et al.*, 1997) and (b) that it occurs after acquisition of CD27 (Vanhecke *et al.*, 1995). CD27 is a member of the tumor necrosis factor receptor (TNFR) family with pro-apoptotic properties (Prasad *et al.*, 1997), its ligand, CD70, has been identified on medullary thymic stromal cells (Hintzen *et al.*, 1995).

In this article, we provide evidence that the transition from the CD4+CD27+CD45RA− to the CD4+CD27+CD45RA+ stage is a major negative

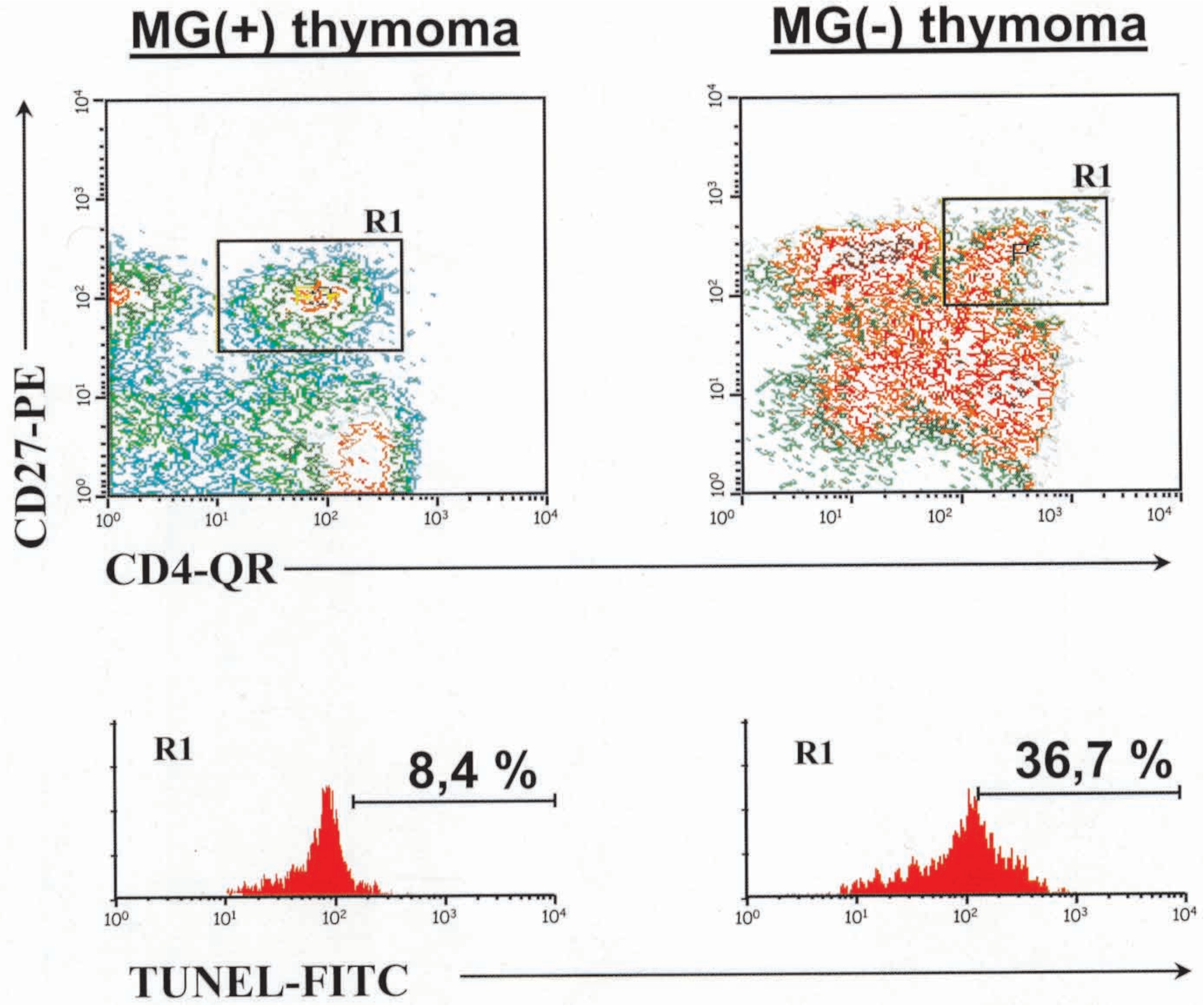

FIGURE 2 Apoptosis of CD4+CD27+ thymocytes is increased in MG(−) thymomas compared to MG(+) thymomas. Representative FACS/TUNEL analysis of a MG(+) and a MG(−)WHO type B2 thymoma showing a significant increase in apoptosis of CD4+CD27+ thymocytes. These findings argue in favour of negative selection/deletion and against precocious release of immature thymocytes from the thymoma.

regulatory event in the shaping of the naive mature CD4+ T cell repertoire in humans. In control thymuses, the percentage of thymocytes dropped by 70% between these two stages. A similar reduction was detected in MG(+) thymomas, especially of the (cortical) WHO subtypes B2 and B3. MG(−) thymomas, by contrast, showed a subtotal loss of CD4+CD27+CD45RA+ cells. TUNEL assays showed that the number of CD4+CD27+ apoptotic cells at this stage was increased compared to MG(+) thymomas, indicating that pro-apoptotic events prevented the development of MG in these patients. Our results are in good agreement with earlier observations that MG(+), but not MG(−) thymomas are enriched for autoreactive T-cells (Sommer *et al.*, 1990; Hoffacker *et al.*, 2000), since only terminally mature CD4+ thymocytes can significantly proliferate after T-cell receptor-mediated signalling (van Hecke *et al.*, 1995).

In the second part of the investigation, we asked whether the observed differences in thymopoiesis between MG(+) and MG(−) thymomas were also pertinent to the CD4+ T cell subset with regulatory functions (regT), since it has been shown that the thymus has the ability to generate regT cells from autoreactive T cells simultaneously with negative selection (Jordan *et al.*, 2001; Kawahata *et al.*, 2002). In line with published data (Stephens *et al.*, 2001), we found CD25 expressed on approximately 10% of CD4+CD8− T cells in control thymuses. By contrast, the percentage of regT cells was statistically significantly reduced in thymomas with only about 3% regT cells in MG(+) thymomas and about 1.3% in MG(−) thymomas (Fig. 3). Interestingly, the percentage of regT cells was strongly reduced also in MG(+) type B2 and B3 (cortical) thymomas, in which terminal thymopoiesis appeared otherwise undisturbed (Fig. 1). This finding indicates that the T cell-stromal interactions that lead to the production of regT cells under physiologic conditions may be disturbed or even missing in thymomas. In thymomas, reduced numbers of regT cells on the one hand are accompanied by increased numbers of autoreactive T cells on the other (Nenninger *et al.*, 1998). It is therefore tempting to speculate that there may be a critical balance between regT and autoreactive

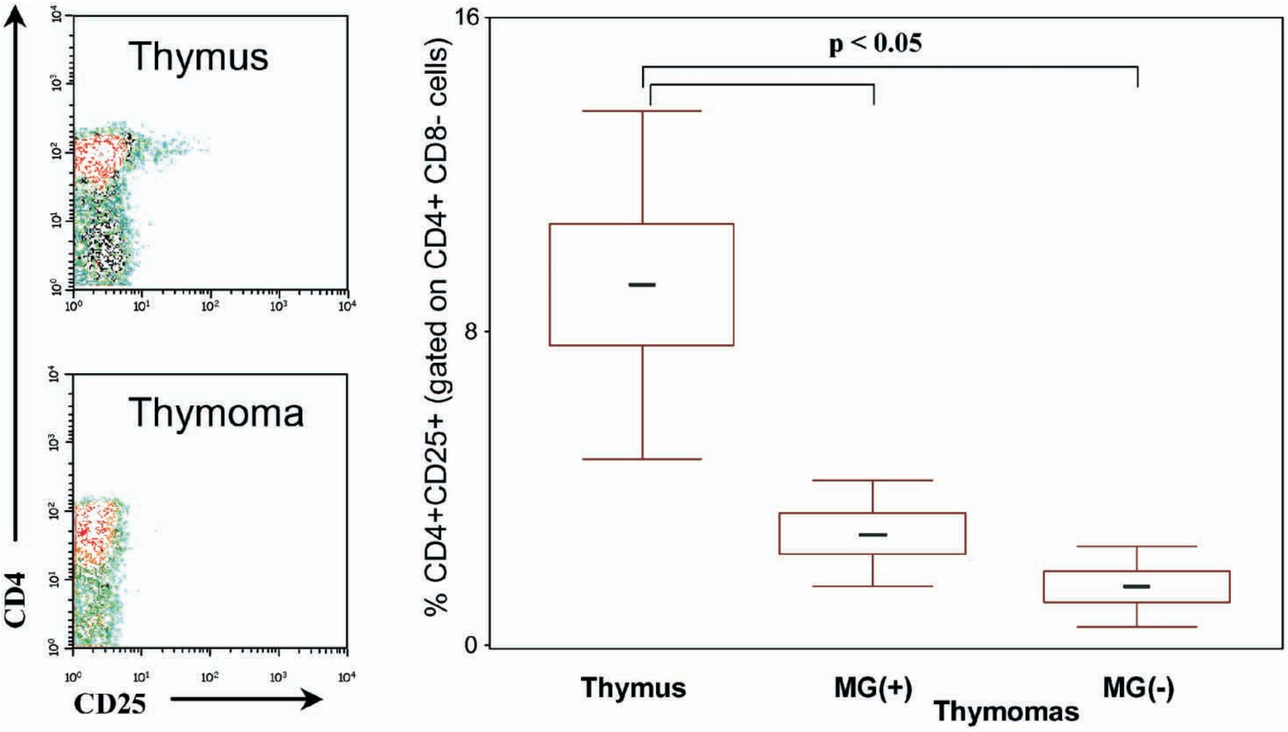

FIGURE 3 Generation of mature T cells with a regulatory (CD4+CD25+) phenotype is highly reduced in thymomas. FACS analysis of 9 MG(+) and 13 MG(−) thymomas as well as 13 non-neoplastic control thymuses showing a statistically significant reduction of CD4+CD25+ regulatory T cells in both MG(+) and MG(−) thymomas, although reduction of this T cell subset was more pronounced in MG(−) tumors.

cells, in which the disturbance of either component may lead to autoimmunity (Fig. 4). Our findings may explain why paraneoplastic MG often deteriorates after thymoma resection (Somnier, 1994; Beeson *et al.*, 1998), since removal of the tumor also implies removal of the small "thymoma-specific" regT cell pool. In MG patients, these regT cells are obviously not sufficient to control autoimmunity, but the clinical course of MG after

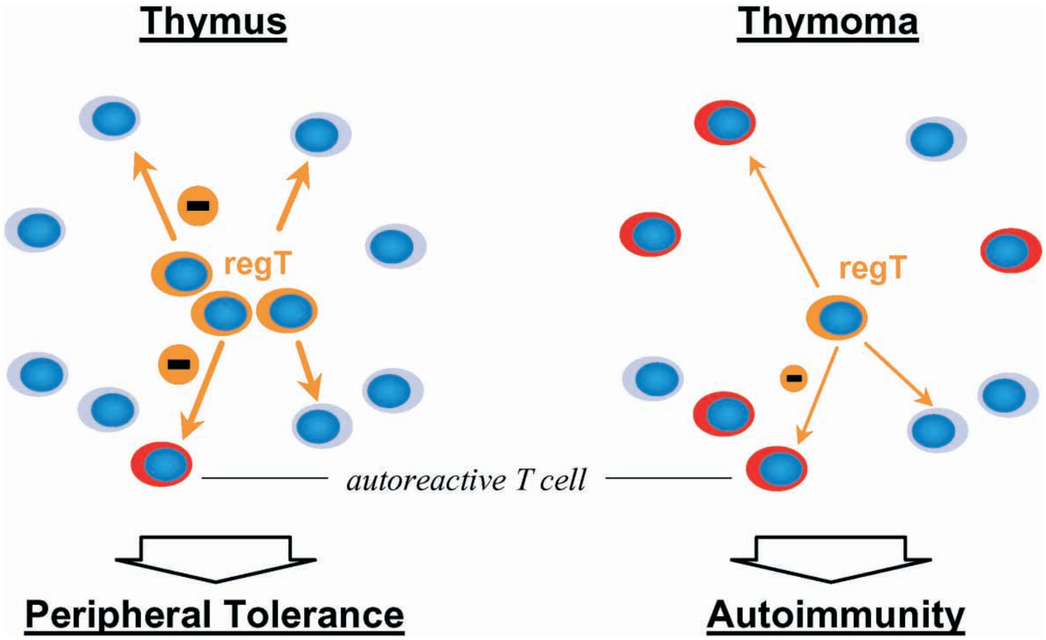

FIGURE 4 Potential consequences of reduced generation of regulatory T cells in the pathogenesis of MG. In the normal thymus, there may be a critical balance between generation of autoreactive and regulatory T cells. Under normal conditions, thymic generation and export of regulatory T cells may suffice to control the few autoreactive T cells present in the blood also of healthy subjects. In thymomas, however, this balance is disturbed by increased numbers of autoreactive T cells and decreased numbers of regulatory T cells. This imbalance may be a contributing factor in breaking peripheral tolerance and in the pathogenesis of autoimmunity.

thymoma resection may indicate that thymoma-derived regT cells indeed have “suppressor” functions.

In summary, we used MG(+) and MG(−) thymomas as powerful human models for the identification of the mechanisms that regulate thymopoiesis and prevent autoimmunity under normal conditions. Our findings indicate that insufficient negative selection may be a pivotal factor in the pathogenesis of MG not only in thymomas, but also in non-neoplastic thymuses with TFH. Moreover, our results highlight the central role of thymoma-derived CD4+ T cells in the pathogenesis of paraneoplastic MG.

### *Acknowledgements*

The authors wish to thank Mrs. Andrea Homburger and Mrs. Isabell Winter for expert technical assistance. P. Ströbel and A. Marx are supported by DFG grant FOR 303/2.

### *References*

Beeson, D., Bond, A.P., Corlett, L., Curnow, S.J., Hill, M.E., Jacobson, L.W., MacLennan, C., Meager, A., Moody, A.M., Moss, P., Nagvekar, N., Newsom-Davis, J., Pantic, N., Roxanis, I., Spack, E.G., Vincent, A. and Willcox, N. (1998) “Thymus, thymoma and specific T cells in myasthenia gravis”, *Ann. NY Acad. Sci.* **841**, 371–387.

Berg, L.J. and Kang, J. (2001) “Molecular determinants of TCR expression and selection”, *Curr. Opin. Immunol.* **13**, 232–241.

Buckley, C., Douek, D., Newsom-Davis, J., Vincent, A. and Willcox, N. (2001) “Mature, long-lived CD4+ and CD8+ T cells are generated by the thymoma in myasthenia gravis”, *Ann. Neurol.* **50**, 64–72.

Douek, D.C., McFarland, R.D., Keiser, P.H., Gage, E.A., Massey, J.M., Haynes, B.F., Polis, M.A., Haase, A.T., Feinberg, M.B., Sullivan, J.L., Jamieson, B.D., Zack, J.A., Picker, L.J. and Koup, R.A. (1998) “Changes in thymic function with age and during the treatment of HIV infection”, *Nature* **396**, 690–695.

Hintzen, R.Q., Lens, S.M., Lammers, K., Kuiper, H., Beckmann, M.P. and van Lier, R.A. (1995) “Engagement of CD27 with its ligand CD70 provides a second signal for T cell activation”, *J. Immunol.* **154**, 2612–2623.

Hoffacker, V., Schultz, A., Tiesinga, J.J., Gold, R., Schalke, B., Nix, W., Kiefer, R., Muller-Hermelink, H.K. and Marx, A. (2000) “Thymomas alter the T-cell subset composition in the blood: a potential mechanism for thymoma-associated autoimmune disease”, *Blood* **96**, 3872–3879.

Hogquist, K.A. (2001) “Signal strength in thymic selection and lineage commitment”, *Curr. Opin. Immunol.* **13**, 225–231.

Itoh, M., Takahashi, T., Sakaguchi, N., Kuniyasu, Y., Shimizu, J., Otsuka, F. and Sakaguchi, S. (1999) “Thymus and autoimmunity: production of CD25+CD4+ naturally anergic and suppressive T cells as a key function of the thymus in maintaining immunologic self-tolerance”, *J. Immunol.* **162**, 5317–5326.

Jordan, M.S., Boesteanu, A., Reed, A.J., Petrone, A.L., Holenbeck, A.E., Lerman, M.A., Naji, A. and Caton, A.J. (2001) “Thymic selection of CD4+CD25+ regulatory T cells induced by an agonist self-peptide”, *Nat. Immunol.* **2**, 301–306.

Kawahata, K., Misaki, Y., Yamauchi, M., Tsunekawa, S., Setoguchi, K., Miyazaki, J. and Yamamoto, K. (2002) “Generation of CD4(+)CD25(+) regulatory T cells from autoreactive T cells simultaneously with their negative selection in the thymus and from nonautoreactive T cells by endogenous TCR expression”, *J. Immunol.* **168**, 4399–4405.

Laufer, T.M., de Koning, J., Markowitz, J.S., Lo, D. and Glimcher, L.H. (1996) “Unopposed positive selection and autoreactivity in mice expressing class II MHC only on thymic cortex”, *Nature* **383**, 81–85.

Laufer, T.M., Fan, L. and Glimcher, L.H. (1999) “Self-reactive T cells selected on thymic cortical epithelium are polyclonal and are pathogenic *in vivo*”, *J. Immunol.* **162**, 5078–5084.

Lindstrom, J.M., Seybold, M.E., Lennon, V.A., Whittingham, S. and Duane, D.D. (1998) “Antibody to acetylcholine receptor in MG: prevalence, clinical correlates, and diagnostic value. 1975 [classical article]”, *Neurology* **51**, 933–939.

MacDonald, H.R., Radtke, F. and Wilson, A. (2001) “T cell fate specification and alphabeta/gammadelta lineage commitment”, *Curr. Opin. Immunol.* **13**, 219–224.

Muller-Hermelink, H.K. and Marx, A. (2000) “Thymoma”, *Curr. Opin. Oncol.* **12**, 426–433.

Naquet, P., Naspetti, M. and Boyd, R. (1999) “Development, organization and function of the thymic medulla in normal, immunodeficient or autoimmune mice”, *Semin. Immunol.* **11**, 47–55.

Nenninger, R., Schultz, A., Hoffacker, V., Helmreich, M., Wilisch, A., Vandekerckhove, B., Hunig, T., Schalke, B., Schneider, C., Tzartos, S., Kalbacher, H., Muller-Hermelink, H.K. and Marx, A. (1998) “Abnormal thymocyte development and generation of autoreactive T cells in mixed and cortical thymomas”, *Lab. Invest.* **78**, 743–753.

Prasad, K.V., Ao, Z., Yoon, Y., Wu, M.X., Rizk, M., Jacquot, S. and Schlossman, S.F. (1997) “CD27, a member of the tumor necrosis factor receptor family, induces apoptosis and binds to Siva, a proapoptotic protein”, *Proc. Natl Acad. Sci. USA* **94**, 6346–6351.

Roncarolo, M.G. and Levings, M.K. (2000) “The role of different subsets of T regulatory cells in controlling autoimmunity”, *Curr. Opin. Immunol.* **12**, 676–683.

Rosai, J. (1999) *Histological Typing of Tumours of the Thymus*, 2nd Edn. (Springer-Verlag, Berlin and Heidelberg).

Rubin, R.L. and Kretz-Rommel, A. (2001) “A nondeletional mechanism for central T-cell tolerance”, *Crit. Rev. Immunol.* **21**, 29–40.

Sakaguchi, S., Sakaguchi, N., Shimizu, J., Yamazaki, S., Sakihama, T., Itoh, M., Kuniyasu, Y., Nomura, T., Toda, M. and Takahashi, T. (2001) “Immunologic tolerance maintained by CD25+CD4+ regulatory T cells: their common role in controlling autoimmunity, tumor immunity, and transplantation tolerance”, *Immunol. Rev.* **182**, 18–32.

Shevach, E.M., McHugh, R.S., Thornton, A.M., Piccirillo, C., Natarajan, K. and Margulies, D.H. (2001) “Control of autoimmunity by regulatory T cells”, *Adv. Exp. Med. Biol.* **490**, 21–32.

Sommer, N., Willcox, N., Harcourt, G.C. and Newsom-Davis, J. (1990) “Myasthenic thymus and thymoma are selectively enriched in acetylcholine receptor-reactive T cells”, *Ann. Neurol.* **28**, 312–319.

Somnier, F.E. (1994) “Exacerbation of myasthenia gravis after removal of thymomas [see comments]”, *Acta Neurol. Scand.* **90**, 56–66.

Stephens, L.A., Mottet, C., Mason, D. and Powrie, F. (2001) “Human CD4(+)CD25(+) thymocytes and peripheral T cells have immune suppressive activity *in vitro*”, *Eur. J. Immunol.* **31**, 1247–1254.

Strobel, P., Helmreich, M., Kalbacher, H., Muller-Hermelink, H.K. and Marx, A. (2001) “Evidence for distinct mechanisms in the shaping of the CD4 T cell repertoire in histologically distinct myasthenia gravis-associated thymomas”, *Dev. Immunol.* **8**, 279–290.

Takeuchi, Y., Fujii, Y., Okumura, M., Inada, K., Nakahara, K. and Matsuda, H. (1995) “Accumulation of immature CD3−CD4+CD8− single-positive cells that lack CD69 in epithelial cell tumors of the human thymus”, *Cell. Immunol.* **161**, 181–187.

van Hecke, D., le Clercq, G., Plum, J. and Vandekerckhove, B. (1995) “Characterization of distinct stages during the differentiation of human CD69+CD3+ thymocytes and identification of thymic emigrants”, *J. Immunol.* **155**, 1862–1872.

van Hecke, D., Verhasselt, B., de Smedt, M., de Paepe, B., Leclercq, G., Plum, J. and Vandekerckhove, B. (1997) “MHC class II molecules are required for initiation of positive selection but not during terminal differentiation of human CD4 single positive thymocytes”, *J. Immunol.* **158**, 3730–3737.

